# Machine learning-based model to predict liposuction outcomes in unilateral breast cancer-related lymphedema

**DOI:** 10.3389/fonc.2026.1916127

**Published:** 2026-08-13

**Authors:** Shuai Pang, Hao Dong, Zhetan Ren, Zhong Liu, Peilin Li, Yonghao Cui, Zixuan Yao, Song Xia, Wenbin Shen

**Affiliations:** Department of Lymphatic Surgery, Capital Medical University Affiliated Beijing Shijitan Hospital; Clinical Center for Lymphatic Disorders, Capital Medical University (CMU), Beijing, China

**Keywords:** breast cancer, liposuction, lymphedema, machine learning techniques, predictive models

## Abstract

**Background:**

Breast cancer-related lymphedema (BCRL) is a common and disabling complication after breast cancer surgery, with substantial effects on limb function and quality of life. Liposuction is an established option for selected patients with chronic BCRL. However, postoperative response is heterogeneous. This study aimed to develop a machine learning model to predict liposuction efficacy in patients with unilateral BCRL.

**Methods:**

We analyzed 623 unilateral BCRL cases undergoing liposuction at Beijing Shijitan Hospital, randomly splitting them 7:3 into training (n=437) and validation (n=186) cohorts. Least absolute shrinkage and selection operator (LASSO) regression with cross-validation guided feature selection. Seven algorithms—logistic regression (LR), support vector machines (SVM), decision trees (DT), artificial neural networks (ANN), LightGBM, XGBoost, and random forests (RF)—were trained and benchmarked. Performance via AUC, calibration, DCA, and Brier score identified SVM as optimal. SHAP interpretation facilitated deployment of a web-based calculator.

**Results:**

The overall rate of favorable outcomes following liposuction was 64.7%. Cross-validated Lasso analysis, factoring in clinical validity and predictive importance, yielded four key predictors: history of erysipelas, preoperative affected-to-unaffected limb volume difference (Preoperative_difference), extracellular water ratio of the affected limb (r-ECW%), and body fat percentage. Among the seven models evaluated, the SVM exhibited the most balanced overall performance, achieving an accuracy of 77.42%, precision of 75.00%, specificity of 90.00%, F1-score of 0.632, Brier score of 0.163, and an AUC of 0.818 (95% CI: 0.757–0.876). Although not possessing the highest AUC, the SVM demonstrated exceptional resistance to overfitting, evidenced by a minimal AUC decrement of merely 0.0298 from the training to the validation set. Both calibration and decision curve analyses corroborated its robust generalizability, underscoring its tangible clinical utility.

**Conclusions:**

An SVM model predicting surgical outcomes in BCRL was created and integrated into a user-friendly online tool. This calculator guides surgical choices based on predictive outputs, offering a valuable reference for refining patient management and treatment plans.

## 1 Introduction

Breast cancer-related lymphedema (BCRL) remains one of the most frequent and burdensome complications after breast cancer surgery. This condition is typically attributed to impaired lymphatic transport capacity following axillary lymph node dissection, radiotherapy, or combined chemoradiotherapy, ultimately leading to the accumulation of lymphatic fluid within the interstitial space (1) Existing epidemiological evidence indicates that over 20% of women develop upper extremity lymphedema following breast cancer treatment; this risk nearly doubles​ when surgery is combined with radiotherapy (2, 3). Early-stage BCRL is typically subtle, occasionally presenting as transient, reversible edema. Its nonspecific nature and symptomatology overlapping with other edematous conditions often lead to misdiagnosis or neglect. Patients commonly present at the clinic only after the development of established complications such as limb hypertrophy, fibrosis, pain, sensory disturbances, functional impairment, or restricted mobility. Ultimately, in advanced disease, progressive trophic alterations—notably elephantiasis, acanthosis, and verrucous hyperplasia—exacerbate physical deformity and functional disability, severely undermining the patient’s long-term well-being (4).

The impact of BCRL extends beyond physical disability. Patients often experience anxiety, depressive symptoms, and dissatisfaction with body image, while the chronic course of the disease creates a substantial and sustained economic burden for both patients and their families (5). As survival after breast cancer continues to improve, the clinical focus has gradually expanded from cancer control alone to long-term survivorship issues. In this context, preventing and detecting BCRL at an early stage remains essential, but improving symptoms, function, and appearance in patients with moderate-to-advanced disease has become equally important.

Liposuction is currently one of the major surgical options for lymphedema and has shown favorable results in patients with middle- to advanced-stage disease who respond poorly to conservative treatment (6). The main objective of liposuction is to remove excessive adipose tissue as well as severely fibrotic skin and subcutaneous tissue, thereby improving local tissue changes and reducing the volume of the affected limb (7). In clinical practice, however, the efficacy of liposuction remains inconsistent. During follow-up, we observed that patients treated during the same period and with similar surgical procedures showed substantial differences in postoperative limb volume reduction. Some patients achieved satisfactory improvement, whereas others still had residual swelling after surgery. This phenomenon suggests considerable interindividual heterogeneity in response to liposuction. However, the factors contributing to suboptimal postoperative outcomes remain poorly elucidated. Current research efforts are predominantly skewed toward the prevention and conservative management of lymphedema, as well as the analysis and prediction of outcomes following lymphovenous anastomosis (LVA). For instance, Wei et al. and Kwan et al. independently developed models for predicting postoperative BCRL risk (8, 9). While instrumental in early identification and risk stratification, these studies predominantly address the risk of lymphedema onset rather than surgical outcomes. Dedicated investigations into liposuction efficacy for BCRL remain scarce; accordingly, a practical and reliable predictive tool is warranted to guide individualized surgical decision-making and identify optimal candidates.

Machine learning provides an effective approach for addressing this clinical problem. Unlike conventional statistical methods, machine learning algorithms excel at managing complex, high-dimensional clinical datasets. By identifying non-linear relationships and variable interactions, these algorithms enhance predictive accuracy and facilitate individualized risk assessment (10, 11). At our institution, eligible patients routinely undergo combined liposuction and lymphaticovenous anastomosis (LVA). Recognizing that some patients harbor apprehension regarding liposuction, this study provides a rationale for tailoring subsequent therapies, specifically redirecting patients with suboptimal liposuction outcomes to standalone LVA or conservative management or secondary liposuction. To our knowledge, this study presents the inaugural machine learning model for predicting liposuction outcomes in BCRL patients. Unlike previous models that focused mainly on the risk of disease occurrence, the present model is directly intended to support surgical decision-making and treatment optimization. In this study, we collected demographic characteristics, medical history, limb volume measurements, and multifrequency bioelectrical impedance parameters from patients with BCRL. Seven machine learning algorithms were developed and compared to predict postoperative liposuction outcomes. In addition, SHAP analysis was used to interpret the optimal model and clarify the contribution of each predictor. Finally, a web-based calculator was developed to facilitate clinical application and provide clinicians with a convenient tool for preoperative assessment.

## 2 Methods

### 2.1 Data source and study population

This retrospective study included patients with breast cancer-related lymphedema (BCRL) who were treated in the Department of Lymphatic Surgery, Beijing Shijitan Hospital, Capital Medical University, between 2019 and 2025. The inclusion criteria were as follows:

1. patients diagnosed with unilateral upper-limb lymphedema secondary to breast cancer surgery, with International Society of Lymphology (ISL) stage II or III disease;
2. patients who underwent isolated liposuction of the affected limb for the first time;
3. patients with complete preoperative data and complete postoperative follow-up data at 3–6 months;
4. patients aged over 18 years.

**Figure 1:**
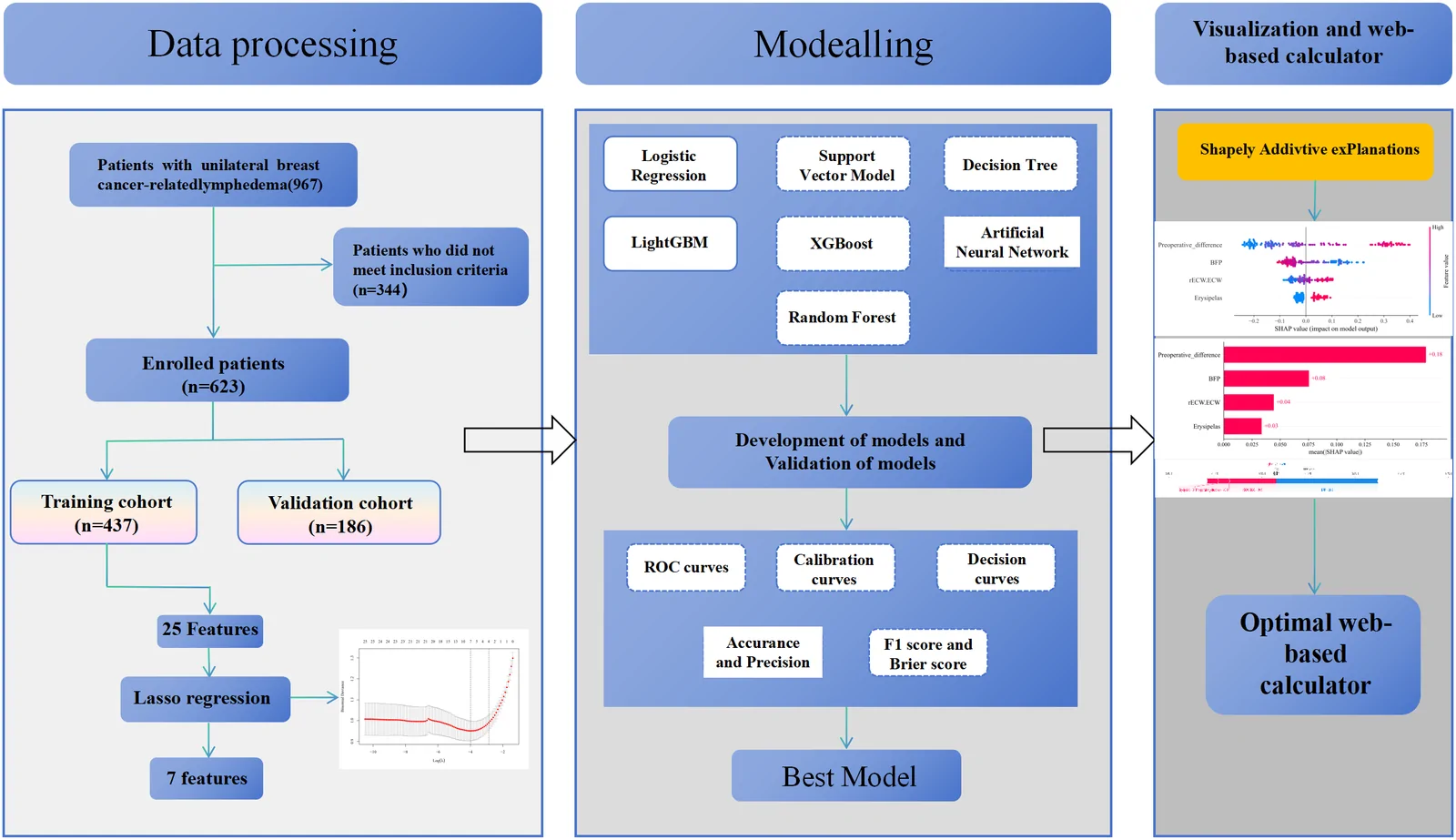
Flowchart outlining patient’s enrollment and study design.

A total of 623 patients were ultimately included in this study. The patient selection process is shown in **Figure 1**. The study protocol was approved by the Ethics Committee of Beijing Shijitan Hospital, Capital Medical University, and was conducted in accordance with the Declaration of Helsinki of 1975, as revised in 1983.

### 2.2 Data collection and sample size

Based on previous studies and clinical experience, 25 clinical variables potentially associated with the efficacy of liposuction in patients with BCRL were collected. These variables included:

1. demographic characteristics: age, height, body weight, and body mass index (BMI);
2. medical history: history of radiotherapy, erysipelas, chemotherapy, latency period, duration of swelling, affected limb side, hypertension, diabetes mellitus, and hyperlipidemia;
3. limb-related measurements: preoperative affected-to-unaffected limb volume difference and lymphedema stage, as well as affected-to-unaffected limb volume difference at 3–6 months after surgery;
4. multifrequency bioelectrical impedance analysis (MFBIA) parameters: extracellular water percentage (ECW%), affected-limb extracellular water percentage (r-ECW%), affected-limb extracellular water volume (r-ECW), affected-limb intracellular water volume (r-ICW), affected-limb extracellular-to-intracellular water ratio, TBF/FFM, body fat percentage, affected-limb impedance values at 1, 5, and 50 kHz, and their ratios relative to the unaffected limb.

For limb volume calculation, commonly used methods include the truncated cone formula, infrared optoelectronic volumetry, and water displacement. In the present study, limb volume was calculated using the truncated cone method (12) (13). All limb measurements were performed by experienced and professionally trained nursing staff.

The sample size of this study was determined in strict accordance with the “10 Events Per Variable (10 EPV)” principle for prediction model development (14). The inclusion of 623 patients, including 220 positive events, fully satisfied the statistical requirements for constructing a machine learning model with four predictors. This provided a robust data foundation for establishing a BCRL liposuction efficacy prediction model with high discriminative ability and a low risk of overfitting.

### 2.3 Outcome definition

Most previous studies have defined upper-limb lymphedema as a relative limb volume increase of >10% or a limb circumference increase of >2 cm. Based on these criteria, the present study evaluated the efficacy of liposuction by measuring the relative limb volume percentage at 3–6 months after surgery. A relative volume increase of ≤10% was defined as a satisfactory therapeutic outcome, whereas a relative volume increase of >10% was defined as a residual swelling outcome (15–17).

### 2.4 Data processing and model development

In this study, variables with missing values ≥20% were excluded (18). The remaining missing data were completed using multiple imputation with the mice package in R. After imputation, the complete dataset was randomly divided into a training cohort (n = 437) and a validation cohort (n = 186) at a ratio of 70:30. The randomization process was performed by computer. Predictor selection and model development were both conducted in the training cohort. To reduce the risk of overfitting, the least absolute shrinkage and selection operator (LASSO) method combined with 10-fold cross-validation was used to identify predictors closely associated with the efficacy of liposuction in patients with BCRL.

In addition, seven machine learning algorithms were applied to predict the postoperative efficacy of liposuction in patients with BCRL: logistic regression (LR), support vector machine (SVM), decision tree (DT), light gradient boosting machine (LightGBM), artificial neural network (ANN), extreme gradient boosting (XGBoost), and random forest (RF). In the training set, model training was performed using hyperparameter grid search optimization and k-fold cross-validation (k = 5). The optimal hyperparameter configuration for each model was determined by grid search (19). Model performance was evaluated in the validation set. The models were assessed from multiple perspectives, including discrimination, calibration, and clinical utility, to identify the optimal model. Discrimination was primarily evaluated using the area under the receiver operating characteristic curve (AUROC). Calibration was assessed using calibration curves and the Brier score, while clinical utility was evaluated using decision curve analysis (DCA). In addition, performance indicators derived from the confusion matrix, including accuracy, sensitivity, specificity, F1 score, and Kappa value, were also used to assess model performance.

Furthermore, the Shapley additive explanations (SHAP) method, a game theory-based interpretability approach proposed by Lundberg, was adopted to explain the output of the machine learning model by calculating SHAP values and evaluating the contribution of each predictor to the model results (20). The final selected optimal model was visually presented and deployed as a web-based tool, enabling convenient use by healthcare professionals.

### 2.5 Statistical analysis

Continuous variables with approximately normal distributions were expressed as mean ± standard deviation (range) and compared using Student’s t test. Continuous variables with non-normal distributions were reported as the median and interquartile range (M (QL, QU)), and compared using the Mann–Whitney U test. Categorical variables were summarized as frequencies and percentages, and comparisons were performed using the chi-square test or Fisher’s exact test, as appropriate. All statistical tests were two-sided, and a P value <0.05 was considered statistically significant. Statistical analyses were performed using SPSS version 27.0 (IBM Corp., Armonk, NY, USA), R version 4.4.0 (R Foundation for Statistical Computing, Vienna, Austria), and Python version 3.12.5 (Python Software Foundation).

## 3 Results

### 3.1 Baseline characteristics

A total of 623 patients with unilateral breast cancer-related lymphedema who underwent liposuction were included in this study, comprising 403 patients (64.7%) in the satisfactory outcome group and 220 patients (35.3%) in the residual swelling group (**Table 1**). In the overall cohort, the median age was 59.00 years [IQR: 52.00, 66.00], and the median body mass index (BMI) was 25.40 kg/m² [IQR: 23.30, 28.25]. The affected limb was on the left side in 337 patients (54.09%) and on the right side in 286 patients (45.91%). The median latency period was 24.00 months [IQR: 12.00, 48.00], and the median duration of swelling was 48.00 months [IQR: 24.00, 96.00]. The median preoperative affected-to-unaffected limb volume difference was 41.97% [IQR: 28.55, 58.39]. Compared with patients in the satisfactory outcome group, those in the residual swelling group were older (62.00 vs. 57.00 years, P < 0.001), had a lower BMI (25.20 vs. 25.60, P = 0.022), a longer duration of swelling (60.00 vs. 48.00 months, P = 0.032), and a greater preoperative limb volume difference (60.39% vs. 35.01%, P < 0.001). The proportion of patients with severe lymphedema was also markedly higher in the residual swelling group (79.55% vs. 37.72%, P < 0.001). With respect to medical history, erysipelas (47.27% vs. 24.81%, P < 0.001), hypertension (37.27% vs. 26.05%, P = 0.005), and diabetes mellitus (20.00% vs. 11.91%, P = 0.009) were more frequently observed in the residual swelling group. In contrast, no significant differences were found between the two groups in terms of radiotherapy, chemotherapy, hyperlipidemia, or latency period (all P > 0.05) (**Table 1**).

**Table 1:** Baseline data of patients.

Variables	Overall (N = 623)	Satisfactory Response (N = 403)	Residual Swelling (N = 220)	*p*
Affected limb side (Left/Right, %)	337/286 (54.09/45.91)	210/193 (52.11/47.89)	127/93 (57.73/42.27)	0.207
Radiotherapy (No/Yes,%)	125/498 (20.06/79.94)	88/315 (21.84/78.16)	37/183 (16.82/83.18)	0.164
Chemotherapy (No/Yes,%)	33/590 (5.30/94.70)	23/380 (5.71/94.29)	10/210 (4.55/95.45)	0.666
Erysipelas (No/Yes,%)	419/204 (67.26/32.74)	303/100 (75.19/24.81)	116/104 (52.73/47.27)	<0.001
Hypertension (No/Yes,%)	436/187 (69.98/30.02)	298/105 (73.95/26.05)	138/82 (62.73/37.27)	0.005
Hyperlipidemia (No/Yes, %)	549/74 (88.12/11.88)	362/41 (89.83/10.17)	187/33 (85.00/15)	0.099
Diabetes (No/Yes,%)	531/92 (85.23/14.77)	355/48 (88.09/11.91)	176/44 (80.00/20.00)	0.009
Preoperative lymphedema stage (Mild to Moderate/Severe,%)	296/327 (47.51/52.49)	251/152 (62.28/37.72)	45/175 (20.45/79.55)	<0.001
Age (median [IQR, years])	59.00 [52.00, 66.00]	57.00 [51.00, 64.00]	62.00 [55.00, 67.00]	<0.001
BMI (median [IQR])	25.40 [23.30, 28.25]	25.60 [23.55, 28.40]	25.20 [22.80, 27.85]	0.022
Latent time (median [IQR], months)	24.00 [12.00, 48.00]	18.00 [12.00, 48.00]	24.00 [12.00, 52.50]	0.081
Swelling time (median [IQR], months)	48.00 [24.00, 96.00]	48.00 [24.00, 84.00]	60.00 [24.00, 108.00]	0.032
Preoperative affected-to-unaffected limb volume difference (median [IQR],%)	41.97 [28.55, 58.39]	35.01 [24.12, 46.12]	60.39 [43.92, 73.30]	<0.001
Affected-limb ECW percentage (median [IQR],%)	40.40 [39.70, 41.20]	40.10 [39.50, 40.75]	41.10 [40.30, 41.92]	<0.001
Affected-limb ECW (median [IQR, L])	0.98 [0.78, 1.23]	0.92 [0.74, 1.10]	1.15 [0.94, 1.42]	<0.001
Affected-limb ICW (median [IQR], L)	1.46 [1.18, 1.76]	1.37 [1.13, 1.61]	1.66 [1.40, 2.00]	<0.001
Affected-limb ECW/ICW ratio (median [IQR])	0.68 [0.66, 0.70]	0.67 [0.65, 0.69]	0.70 [0.67, 0.72]	<0.001
TBW. FFM (Mean ± SD,%)	73.98 ± 0.38	73.91 ± 0.18	74.13 ± 0.26	<0.001
X1kHz.ratio (median [IQR])	235.70 [195.55, 284.80]	257.00 [217.00, 300.85]	203.55 [171.62, 246.05]	<0.001
X1kHz.ratio (median [IQR])	1.61 [1.38, 1.90]	1.49 [1.31, 1.71]	1.87 [1.60, 2.12]	<0.001
X5kHz.ratio (median [IQR])	232.30 [192.75, 280.75]	253.20 [213.70, 297.50]	198.80 [169.25, 242.18]	<0.001
X5kHz.ratio (median [IQR])	1.61 [1.38, 1.88]	1.49 [1.30, 1.69]	1.87 [1.61, 2.11]	<0.001
X50kHz.ratio (median [IQR])	218.60 [181.20, 263.90]	239.10 [201.30, 277.10]	188.30 [159.17, 227.75]	<0.001
X50kHz.ratio (median [IQR])	1.55 [1.34, 1.83]	1.45 [1.27, 1.65]	1.80 [1.55, 2.06]	<0.001
Body fat percentage (median [IQR],%)	34.80 [30.90, 38.35]	35.70 [32.70, 38.90]	33.05 [28.20, 37.02]	<0.001

Multifrequency bioelectrical impedance analysis showed that the residual swelling group had a higher affected-limb extracellular water percentage (41.10% vs. 40.10%, P < 0.001). The affected-limb extracellular water volume (1.15 vs. 0.92 L), intracellular water volume (1.66 vs. 1.37 L), and ECW/ICW ratio (0.70 vs. 0.67) were also significantly higher in this group (all P < 0.001). In addition, TBW/FFM was higher in the residual swelling group (74.13 ± 0.26 vs. 73.91 ± 0.18, P < 0.001), whereas body fat percentage was lower (33.05% vs. 35.70%, P < 0.001). The absolute impedance values of the affected limb at 1, 5, and 50 kHz were lower in the residual swelling group, while the corresponding affected-to-unaffected limb impedance ratios were higher (all P < 0.001)(**Table 1**).

Taken together, patients with poor postoperative outcomes tended to be older, had a longer disease course of swelling, a larger preoperative limb volume difference, and a higher proportion of severe lymphedema. They were also more likely to have concomitant erysipelas, hypertension, and diabetes mellitus. Their bioelectrical impedance profiles suggested more pronounced fluid retention and lower fat content, features that were closely associated with suboptimal efficacy after liposuction.

### 3.2 Model development

**Figure 2:**
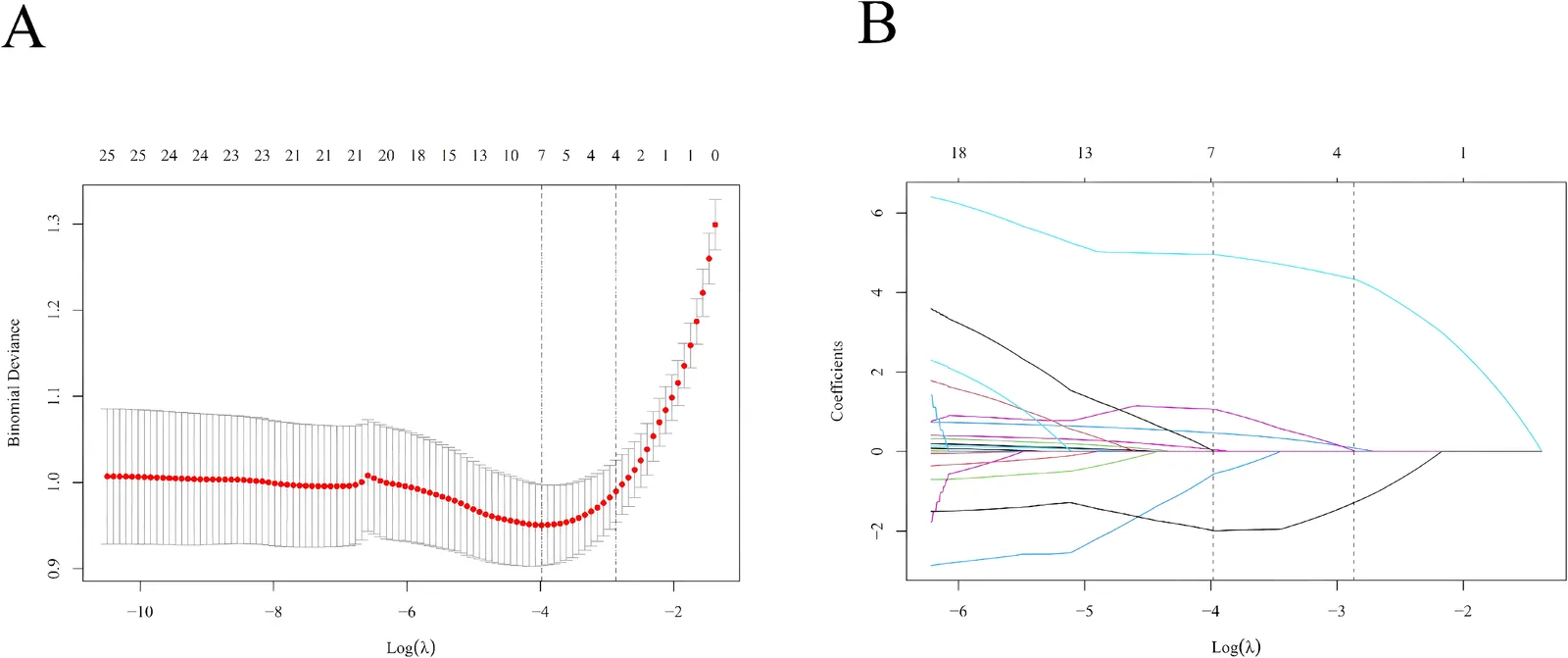
Presentation of the results of the LASSO regression analysis. **(A)** LASSO Regression Model Factor Selection: The left dashed line represents the optimal lambda value (λ·min), while the right dashed line marks the lambda value within one standard error of the optimal (lambda.1se); **(B)** LASSO regression model screening variable trajectories.

Feature selection was performed using least absolute shrinkage and selection operator (LASSO) regression with tenfold cross-validation to determine the optimal regularization parameter λ. The final model retained four predictors selected at λ = 1, after clinical plausibility assessment and evaluation of incremental predictive contribution. **Figure 2A** illustrates the relationship between cross-validated mean squared error and the log(λ) penalty term, and **Figure 2B** shows the coefficient paths of candidate variables, indicating the point at which coefficients shrink to zero (**Figure 2**.).

The four selected predictors were: history of erysipelas, preoperative affected-to-unaffected limb volume difference, extracellular water percentage of the affected limb (r-ECW%), and body fat percentage. LASSO coefficients confirmed that erysipelas, larger preoperative volume difference, and elevated r-ECW% were associated with increased risk of unsatisfactory outcome, whereas higher body fat percentage conferred a protective effect. These four variables were harnessed as input features to establish prediction models via seven distinct machine learning algorithms, encompassing LR, DT, RF, XGBoost, ANN, LightGBM, and SVM. Model training employed fivefold cross-validation with grid search for hyperparameter tuning to minimize overfitting and improve generalizability. The optimal hyperparameter configurations for each algorithm are provided in **Table 2**.

**Table 2:** Optimal parameters for each model.

Full super-para meters of techniques
Decision Tree (DT):
DT Classifer: {‘ccp_alpha’: 0.001, ‘max_depth’: 5, ‘max_features’: ‘sqrt’, ‘min_samples_leaf’: 15, ‘min_samples_split’: 10}
Random Forest (RF):
RF Classifer: n_estimators = 300, max_features = sqrt
eXtreme Gradient Boosting (XGBoost):
XGBoost Classifer: {‘colsample_bytree’: 0.8, ‘gamma’: 0.1, ‘learning_rate’: 0.01, ‘max_depth’: 3, ‘n_estimators’: 150, ‘reg_alpha’: 0.5, ‘reg_lambda’: 1, ‘subsample’: 0.6}
Light Gradient Boosting Machine (LightGBM):
LightGBM Classifer: {‘subsample’: 0.8, ‘reg_lambda’: 1, ‘reg_alpha’: 0.5, ‘num_leaves’: 15, ‘n_estimators’: 100, ‘min_child_samples’: 50, ‘learning_rate’: 0.05, ‘colsample_bytree’: 0.7}
Support Vector Machine (SVM):
SVM Classifer: {‘C’: 0.1, ‘degree’: 2, ‘gamma’: ‘scale’, ‘kernel’: ‘linear’}
Artificial Neural Network (ANN):
ANN Classifer:{‘activation’: ‘relu’, ‘alpha’: 0.1, ‘hidden_layer_sizes’: (50, 50), ‘learning_rate_init’: 0.001}

### 3.3 Model validation

**Figure 3:**
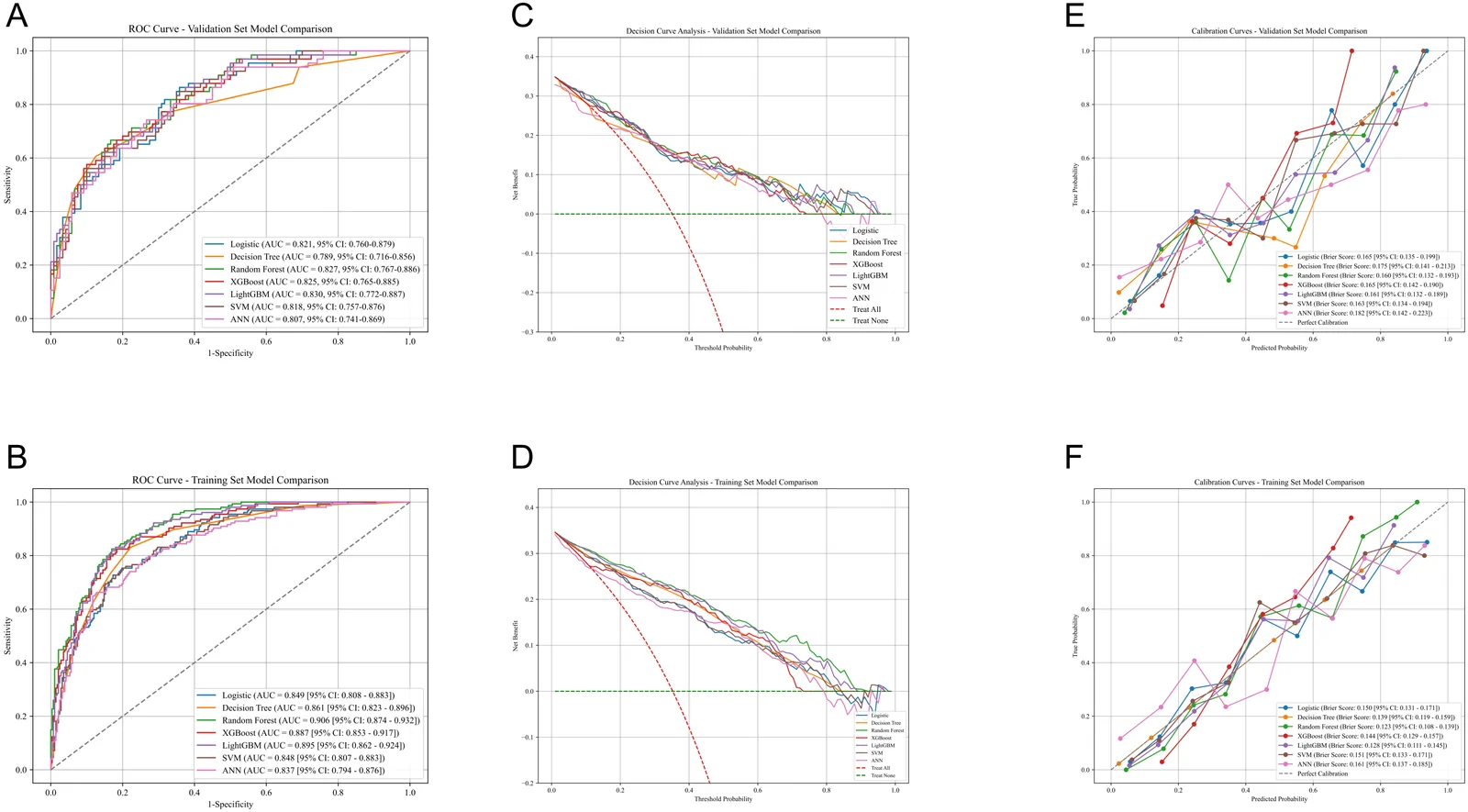
The performance and comparison of seven different predictive models. **(A)** ROC Curves for the Validation Set; **(B)** ROC Curves for the Training Set; **(C)** Decision Curve Analysis for the Validation Set; **(D)** Decision Curve Analysis for the Training Set; **(E)** Calibration Curve for the Validation Set; **(F)** Calibration Curve for the Training Set.

Seven models were evaluated for their ability to predict liposuction efficacy in BCRL. The comparative performance of the machine learning models is presented in **Table 3** and **Figure 3**. **Table 3** delineates the AUC, accuracy, sensitivity, specificity, positive predictive value (PPV), negative predictive value (NPV), and F1-score for all seven algorithms. In the training cohort, AUC values ranged from 0.837 to 0.906. In the validation cohort, the SVM model demonstrated the most balanced and robust performance: AUC = 0.818 (95% CI: 0.757–0.876), accuracy = 0.774 (tied with XGBoost), precision = 0.750, specificity = 0.900, F1 score = 0.632, and Kappa = 0.475. Although Random Forest achieved the highest AUC in both the training (0.906) and validation (0.827) sets, its absolute reduction of 0.079 was among the most pronounced across all models, suggesting potential overfitting. In contrast, SVM exhibited the smallest decline (0.0298) from training to validation, demonstrating optimal generalizability and stability. The calibration curve demonstrated good agreement between the predicted probabilities by the SVM model and the observed outcomes. Furthermore, decision curve analysis (DCA) confirmed that the SVM model provided a stable net clinical benefit across a wide range of threshold probabilities, underscoring its reliability for clinical application. Based on discrimination, calibration, stability, and clinical net benefit, the SVM model was selected as the optimal predictor.

**Table 3:** Detailed performance metrics on the training and validation sets for various machine learning models predicting the efficacy of liposuction surgery in BCRL patients.

Cohort	Performance metrics	Logistic	Decision tree	Random forest	XGBoost	LightGBM	SVM	ANN
	AUC	0.849387	0.861365	0.905879	0.887465	0.895071	0.847942	0.837272
	95% CI Lower	0.80801	0.823488	0.874368	0.852774	0.861561	0.806959	0.793739
	95% CI Upper	0.882817	0.895812	0.932049	0.916908	0.92407	0.883054	0.876434
	Accuracy	0.775744	0.798627	0.814645	0.798627	0.812357	0.778032	0.796339
	Precision	0.725806	0.70625	0.766423	0.794643	0.764706	0.72093	0.733813
Training	Sensitivity	0.584416	0.733766	0.681818	0.577922	0.675325	0.603896	0.662338
	Specificity	0.879859	0.833922	0.886926	0.918728	0.886926	0.872792	0.869258
	F1 Score	0.647482	0.719745	0.721649	0.669173	0.717241	0.657244	0.696246
	Kappa	0.485845	0.562692	0.583423	0.529567	0.577638	0.495002	0.543665
	Youden’s J	0.464274	0.567688	0.568744	0.49665	0.56225	0.476688	0.531596
	PPV	0.725806	0.70625	0.766423	0.794643	0.764706	0.72093	0.733813
	NPV	0.795527	0.851986	0.836667	0.8	0.833887	0.801948	0.825503
	AUC	0.820581	0.788636	0.826768	0.824747	0.830114	0.818182	0.807197
	95% CI Lower	0.759945	0.716454	0.766569	0.764994	0.772251	0.757171	0.741243
	95% CI Upper	0.878844	0.856132	0.88591	0.884628	0.886696	0.875531	0.868532
	Accuracy	0.758065	0.741935	0.763441	0.774194	0.763441	0.774194	0.747312
	Precision	0.698113	0.628571	0.703704	0.76087	0.689655	0.75	0.661017
Validation	Sensitivity	0.560606	0.666667	0.575758	0.530303	0.606061	0.545455	0.590909
	Specificity	0.866667	0.783333	0.866667	0.908333	0.85	0.9	0.833333
	F1 Score	0.621849	0.647059	0.633333	0.625	0.645161	0.631579	0.624
	Kappa	0.447087	0.443946	0.461295	0.470732	0.468847	0.474576	0.434614
	Youden’s J	0.427273	0.45	0.442424	0.438636	0.456061	0.445455	0.424242
	PPV	0.698113	0.628571	0.703704	0.76087	0.689655	0.75	0.661017
	NPV	0.781955	0.810345	0.787879	0.778571	0.796875	0.782609	0.787402

### 3.4 Web-based calculator implementation

Based on the optimal SVM model, we deployed a web-based calculator via the Streamlit framework to predict liposuction outcomes in BCRL patients. Accessible at (https://bcrlpredictor-5q8kwapbvaev4ved8mrr63.streamlit.app/), the tool generates outcome probabilities upon entry of measured clinical parameters, thereby facilitating preoperative assessment and guiding subsequent therapeutic strategies. Boasting operational simplicity and clear reporting, the web-tool delivers a streamlined, efficient predictive solution for both healthcare providers and patients. 

### 3.5 SHAP-based model interpretation

**Figure 4:**
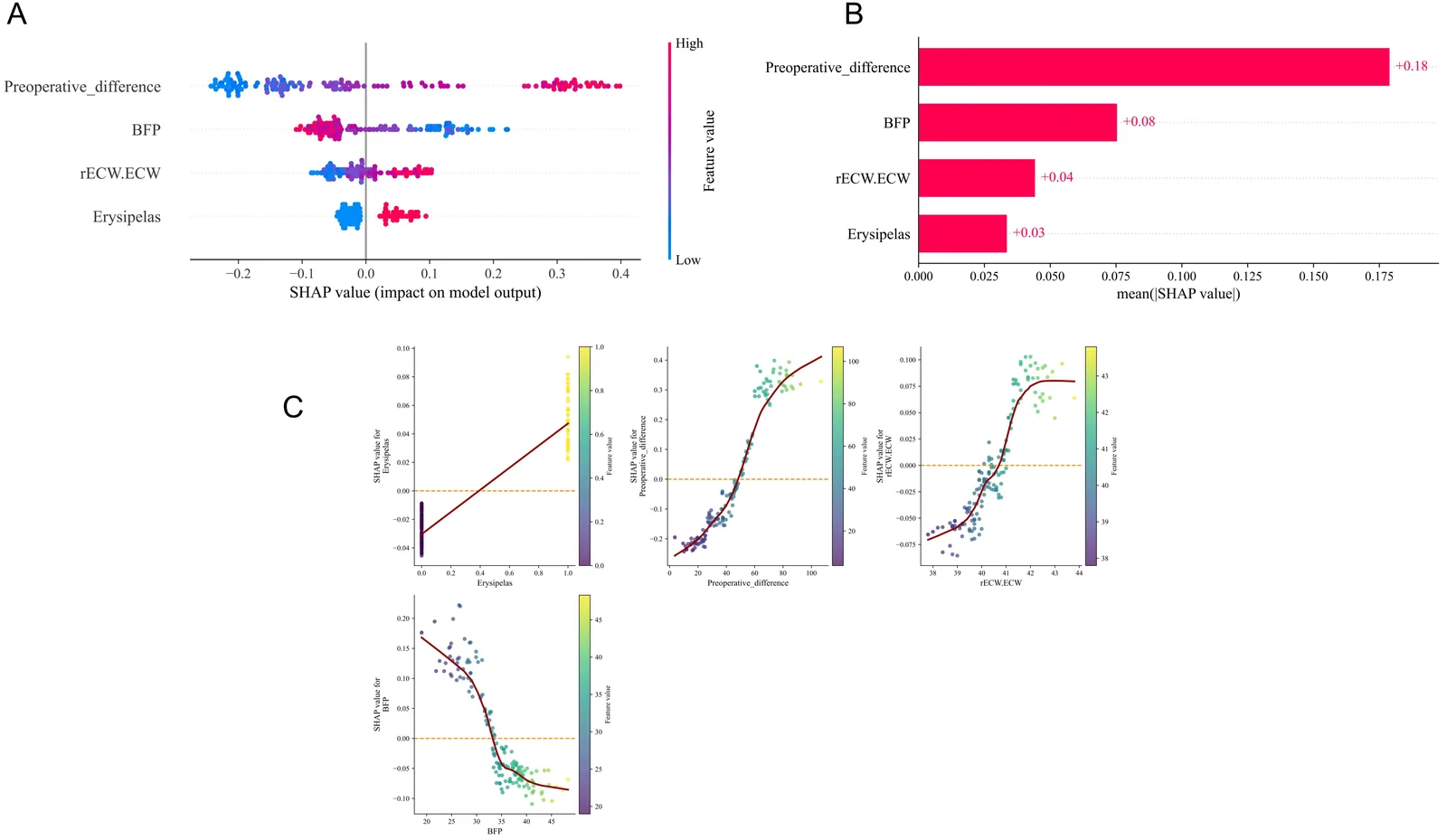
SHAP of the model. **(A)** Characteristic attributes in SHAP. The abscissa is the SHAP value, and each line denotes a feature. Higher eigenvalues are indicated by red dots, and lower eigenvalues are indicated by blue dots. **(B)** Feature importance ranking plot for the random forest model. **(C)** Dependence plot: The relationship between the values of features and their corresponding SHAP values. The abscissa represents the feature value, the ordinate represents the SHAP value, and the red curve is the locally weighted regression line, reflecting the overall trend between the feature and SHAP values.

**Figure 5:**
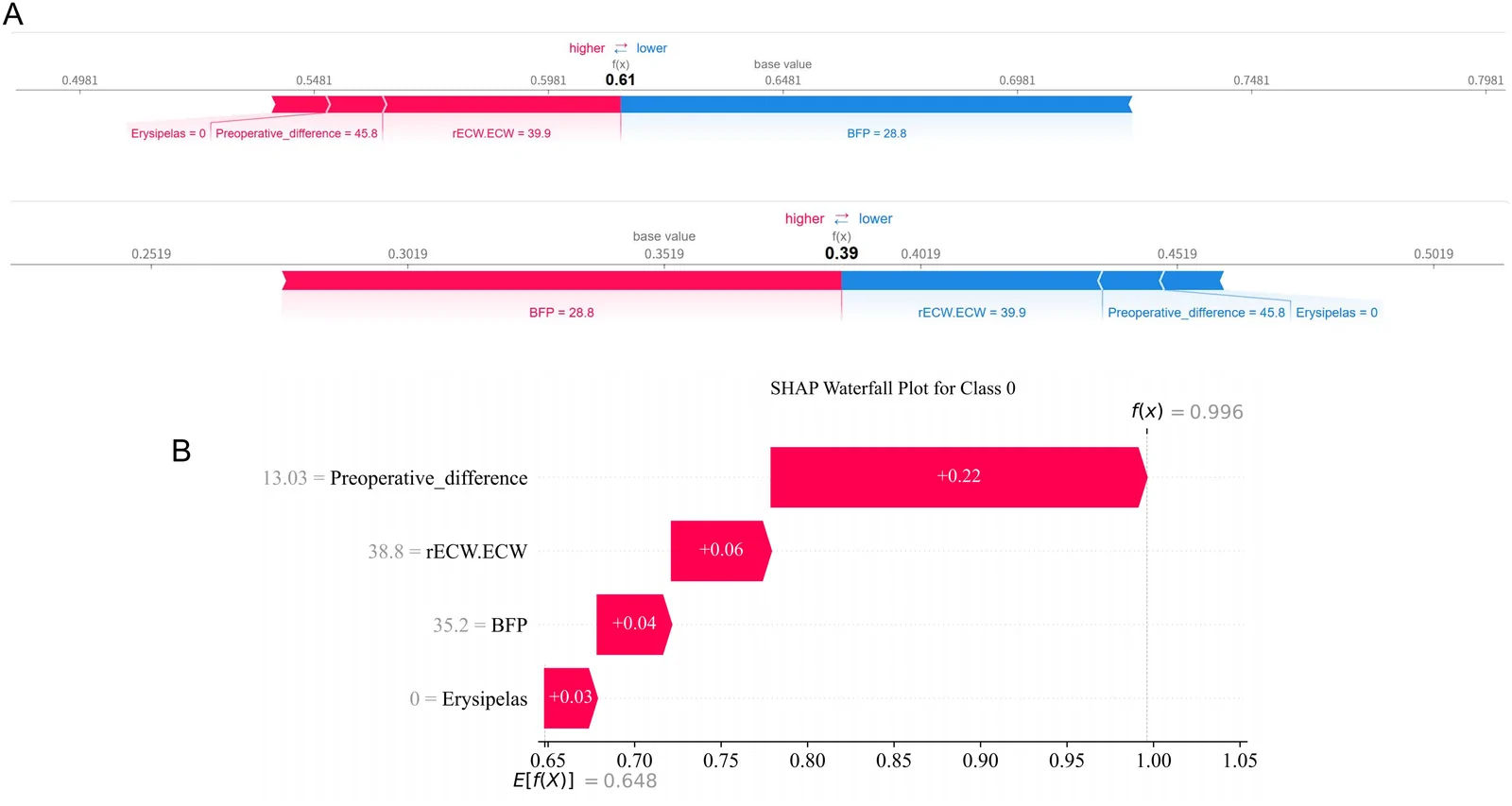
SHAP individual prediction explanations for the SVM model. **(A)** Force plot: red colour represented a positive influence of the feature on satisfactory outcome. Blue colour represented the opposite. **(B)** Waterfall plot: It was an extended form of force plot.

To elucidate the contribution of the selected variables, we employed SHAP summary plots for visualization. a SHAP beeswarm plot, depicting the impact of each feature on the model output—the efficacy of liposuction. The X-axis (SHAP value) quantifies each feature’s contribution; higher values indicate a stronger association with suboptimal surgical outcomes, whereas lower values suggest a protective effect. The color gradient denotes feature magnitude, with red indicating high values and blue indicating low. Notably, all predictors exerted a marked influence on the outcome. Specifically, a higher body fat percentage correlated with improved efficacy, while greater preoperative limb volume discrepancy, elevated extracellular water ratio (r-ECW%), and a history of erysipelas were associated with diminished efficacy. **Figure 4B**. ranks variables by mean SHAP value, confirming that preoperative volume difference is the most influential predictor. These findings are consistent with the direction and magnitude of associations observed in the LASSO regression (**Figure 4C**) and support the clinical plausibility of the model. Finally, individual-level interpretability for a representative case is presented in **Figures 5A**, **B**.

## 4 Discussion

In this study, a machine learning-based model was developed and validated to predict liposuction efficacy in patients with breast cancer-related lymphedema (BCRL). LASSO regression initially identified seven variables with non-zero coefficients. After further assessment of clinical plausibility and predictive contribution, four key predictors were retained from 25 candidate variables. Among the seven candidate models, the support vector machine (SVM) model showed the most favorable overall performance, with an area under the receiver operating characteristic curve (AUC) of 0.818, indicating good discriminative ability. SHAP-based interpretability analysis further supported the contribution of these variables to model predictions.

Globally, the incidence of breast cancer has continued to increase, and breast cancer is now the most commonly diagnosed malignancy worldwide (21). In China, the incidence of female breast cancer has also risen steadily, with an increase exceeding the global average, while mortality has remained relatively stable as diagnostic and therapeutic systems have improved (22). As one of the most common and disabling complications after breast cancer treatment, BCRL is therefore becoming increasingly prevalent. Accordingly, prevention, early detection, and treatment of BCRL have received growing attention both in China and internationally. Current research on BCRL has largely focused on prevention, conservative management, and physiologic surgical procedures. In contrast, evidence related to liposuction, particularly prediction of postoperative response, remains limited. The present model was therefore developed to estimate liposuction efficacy and to help clinicians identify patients at higher risk of unsatisfactory response before surgery. Such prediction may facilitate timely adjustment of therapeutic strategies and reduce the likelihood of ineffective surgical intervention.

Using LASSO regression with tenfold cross-validation, followed by assessment of clinical relevance and predictive contribution, four predictors associated with liposuction outcomes in BCRL were identified. Higher preoperative affected-to-unaffected limb volume difference, elevated r-ECW%, and previous erysipelas increased the risk of poor liposuction response, whereas higher body fat percentage was associated with better postoperative efficacy. A larger preoperative limb volume difference indicates more severe limb swelling and was linked to poorer surgical outcomes, which is consistent with previous observations. In a long-term cohort study, Penn et al. reported that a greater initial limb circumference discrepancy at the onset of swelling, along with the maximum discrepancy during follow-up, served as independent predictors of persistent BCRL. This implies that more severe swelling correlates with a poorer response to conservative treatment; notably, however, this study did not investigate surgical outcomes (23). From a clinical perspective, greater interlimb differences in circumference and volume provide relatively direct indications of lymphedema progression. Compared with patients exhibiting smaller differences, those with greater interlimb circumference and volume differences often present with non-pitting edema and may even develop trophic skin changes, suggesting a more advanced stage of lymphedema. From a pathophysiological perspective, compared with other indirect variables, interlimb volume difference more directly reflects the overall disease burden of the affected limb. A greater preoperative interlimb volume difference generally indicates progression to a more advanced stage of disease, characterized by more severe lymphatic vessel damage, accumulation of protein-rich interstitial fluid, and extensive adipose tissue deposition. Although liposuction can effectively remove hypertrophic adipose and fibrotic tissues, it cannot restore impaired lymphatic transport function. Consequently, these patients are inherently at greater risk of residual swelling, corresponding to an increased likelihood of suboptimal surgical outcomes (24).

Multifrequency bioelectrical impedance analysis (MFBIA) is a routinely employed clinical modality for assessing human body composition. This technique enables the sensitive quantification of extracellular water, intracellular water, total body water, and fat mass (25). Prior investigations demonstrate that MFBIA parameters, particularly extracellular water volume and extracellular water fraction of the affected limb, correlate positively with limb volume changes. Given their enhanced accuracy over traditional anthropometric measurements, these markers hold promise as refined tools for both lymphedema diagnosis and surgical guidance (26, 27). Yasunaga et al. reported that both extracellular water and intracellular water in the affected limb increase after lymphedema onset, and that lymphovenous anastomosis (LVA) can reduce both components. They also found that changes in extracellular and intracellular water volumes were comparable both at disease onset and after LVA. In addition, the extracellular water percentage of the affected limb may reflect disease duration and severity in both upper- and lower-extremity lymphedema (28). These findings indicate that MFBIA has been increasingly applied in the diagnosis and severity assessment of lymphedema. In the present study, r-ECW% may be associated with fluid retention and persistent lymphatic dysfunction. Compared with age, disease duration, and comorbidities, this parameter may provide a more direct reflection of the real-time fluid status of the affected limb before surgery. Previous studies have shown that a higher extracellular water percentage generally indicates more severe limb swelling and greater interstitial fluid retention. Because liposuction primarily reduces the adipose and other solid tissue components of chronic lymphedema but does not directly restore damaged lymphatic drainage pathways, patients whose swelling is predominantly attributable to fluid retention may be more likely to experience residual postoperative swelling, thereby limiting the effectiveness of liposuction.

Previous studies have demonstrated that a history of erysipelas is a risk factor for the development of lymphedema, and likewise, a risk factor for suboptimal outcomes following liposuction. Research by Li et al. confirmed that a history of erysipelas is an established risk factor for lymphedema and provided new insights into the molecular association between erysipelas and lymphedema recurrence. Their findings indicated that erysipelas may jointly promote adipose tissue deposition, chronic inflammation, and tissue fibrosis by upregulating multiple signaling pathways, including the PI3K/AKT, TGF-β, and p38 MAPK pathways, thereby aggravating lymphatic dysfunction and increasing the risk of postoperative recurrence. For example, excessive activation of the PI3K/AKT pathway in erysipelas lesions may induce abnormal adipose tissue proliferation, intensify mechanical compression of lymphatic vessels, and impair interstitial fluid drainage. Overexpression of TGF-β may further exacerbate these effects by triggering fibroblast activation, collagen deposition, and extracellular matrix stiffening. Ultimately, these changes impede lymphatic drainage and compromise tissue repair after liposuction. Activation of the p38 MAPK pathway may promote inflammatory cell infiltration, reactive oxygen species (ROS) accumulation, and increased expression of matrix-degrading enzymes, thereby further impairing lymphatic function and aggravating fluid drainage obstruction. Therefore, impaired lymphatic return reduces local immune clearance capacity, making patients more susceptible to erysipelas. Erysipelas, in turn, worsens the underlying lymphatic dysfunction, creating a vicious cycle of recurrent erysipelas and lymphedema. Such recurrence elevates surgical complexity and compromises the volumetric outcomes of liposuction (29). Here, erysipelas history functioned as a marker for systemic inflammation and metabolic abnormalities, and we similarly demonstrated its correlation with poor outcomes. These findings emphasize the importance of active prevention and management of erysipelas in patients with BCRL. Patient education regarding skin care, treatment of tinea pedis, avoidance of skin trauma, and early infection control should be strengthened to improve surgical outcomes and reduce postoperative complications.

An important finding of this study was the association between higher body fat percentage and satisfactory liposuction efficacy. On the surface, this finding appears to contradict the long-standing recognition of obesity as a risk factor for BCRL incidence and progression—given that obese patients typically present with higher body fat percentages—yet the two reflect distinct dimensions of the problem. Previous studies have shown that obesity can promote lymphedema through chronic low-grade inflammation, increased lymphatic load, impaired lymphatic contractility, and abnormal adipose tissue deposition (30). Following the onset of lymphedema, persistent lymphatic stasis triggers fat accumulation, fibrosis, and inflammatory tissue remodeling, leading to a gradual pathological shift from fluid-dominant swelling to fibrofatty hyperplasia. Liposuction primarily targets this aberrant subcutaneous and fibrofatty tissue, rather than directly reconstructing the lymphatic drainage system. Therefore, for patients with established chronic BCRL contemplating liposuction, a higher body fat percentage may signify a greater abundance of adipose or fibrofatty tissue components within the edematous limb—tissue that is readily amenable to suction removal, potentially yielding more pronounced volume improvement. Conversely, patients with lower body fat percentage may have had less removable adipose tissue, and their residual swelling may have been driven more by interstitial fluid retention, fibrosis, or intrinsic lymphatic drainage impairment, resulting in relatively poorer response to liposuction.

This finding should not be interpreted as evidence that patients with a high body fat percentage protects against BCRL, nor does it suggest that obesity do not require weight management. Obesity remains an important risk factor for the development and progression of BCRL and should be addressed through long-term follow-up and weight-control strategies (31). The protective role of body fat percentage in the model more likely reflects the extent of resectable target tissue components amenable to liposuction. Future studies incorporating magnetic resonance imaging, ultrasonography, indocyanine green lymphography, or localized tissue composition analysis are needed to further clarify the relationship between body fat percentage and liposuction response, as well as the underlying mechanisms.

In summary, compared with relatively indirect candidate features such as age, disease duration, and other comorbidities, the four predictors selected in this study may more directly characterize the local disease burden, fluid and adipose tissue composition, and inflammation-related lymphatic injury that influence the efficacy of liposuction. Some variables not retained in the final model may exert their effects through indicators that more closely reflect the disease phenotype or may contain overlapping information. For example, a higher body mass index may overlap with body fat percentage, while other MFBIA-derived parameters, such as extracellular water percentage (ECW%), intracellular water content in the unaffected and affected limbs, and bioelectrical impedance measurements of the unaffected and affected limbs, may overlap with the extracellular fluid ratio of the affected limb (r-ECW%). Therefore, the variable selection results of this study should be interpreted as indicating that these parameters have relatively high independent predictive contributions within the current dataset, rather than as evidence of a definitive causal relationship with postoperative outcomes.

Machine learning has been increasingly used to develop prediction models for lymphedema, particularly for estimating the risk of BCRL or lower-limb lymphedema after gynecologic cancer surgery. For example, Wei et al. selected 24 lymphedema-related symptoms as candidate predictors and established an early symptom warning model for BCRL (32). However, studies predicting the therapeutic response to liposuction in patients with established BCRL remain scarce. The present study therefore addresses an important gap in the field. By incorporating Support Vector Machines (SVM) and other machine learning algorithms, this study not only enhanced predictive performance but also elucidated the key factors influencing liposuction outcomes. In addition, a web-based application was developed to facilitate clinical use of the final model.

The present model should be positioned as an adjunctive tool for preoperative risk stratification and shared decision-making rather than as a stand-alone instrument for determining the indications or contraindications for liposuction. In clinical practice, after completing the routine evaluation, surgeons may enter the patient’s history of erysipelas, preoperative volume difference between the affected and unaffected limbs, r-ECW% of the affected limb, and body fat percentage into the web-based calculator to estimate the probability of postoperative residual swelling. The model output should then be interpreted comprehensively in conjunction with the duration and clinical stage of lymphedema, degree of pitting, extent of adipose tissue hypertrophy and fibrosis, response to conservative treatment, tissue composition and residual lymphatic drainage function on imaging, as well as the patient’s infection status, comorbidities, anesthetic risk, and adherence to postoperative compression therapy.

For patients with a low model-predicted risk who have clinically and radiologically confirmed adipose or fibroadipose tissue hypertrophy and persistent substantial limb enlargement despite standardized conservative treatment, the model output may provide additional support for selecting liposuction. Conversely, patients predicted to be at high risk of residual swelling should not be excluded from liposuction solely on the basis of the predicted risk; instead, the underlying sources of risk should be identified. For example, a higher r-ECW% may indicate a greater component of fluid retention, a larger preoperative volume difference may reflect a greater disease burden, and a history of erysipelas may indicate increased risks of local inflammation and infection. Such patients may undergo further evaluation using ultrasonography, magnetic resonance imaging, indocyanine green lymphography, or other appropriate examinations to characterize local tissue composition and lymphatic drainage function. The feasibility of intensified conservative treatment, physiologic lymphatic surgery, liposuction, or combined or staged treatment may then be considered and discussed. Furthermore, the exclusion of certain variables from the final model does not imply that they lack clinical relevance. Factors including age, hypertension, diabetes mellitus, oncologic status, cardiopulmonary function, coagulation status, risk of venous thromboembolism, active infection, and adherence to postoperative compression therapy remain essential when assessing surgical safety and long-term outcomes. Variable selection in the model reflects the independent predictive contribution of each variable to the outcome investigated in this study and cannot replace a comprehensive preoperative evaluation. Given that the model has not yet undergone external validation, its output is currently more appropriate for risk communication and as an aid to clinical judgment. It should not be used for automated treatment decision-making or as the sole basis for excluding patients from liposuction. The tool is simple and accessible, and may support preoperative counseling, shared decision-making, and selection of appropriate therapeutic strategies for patients with BCRL.

This study has several limitations. First, although 25 variables were analyzed, some potentially relevant clinical factors were not included. In particular, important oncological and axillary treatment variables, such as the type of axillary surgery, number of lymph nodes removed, extent of lymph node involvement, and history of prophylactic lymphatic surgery, may affect lymphatic dysfunction and the response to liposuction. However, because this was a retrospective study based on clinical and treatment records and perioperative follow-up data from patients with BCRL, these variables could not be consistently retrieved, and complete standardized data were not available for all patients. To avoid unstable estimates and potential bias resulting from extensive data imputation, these variables were excluded from the final model. Future studies should incorporate these factors and evaluate their potential confounding effects. Second, because external validation was not performed in this study, the generalizability of the model to other cohorts needs to be confirmed in future research.

## 5 Conclusions

This study developed and validated a machine learning-based model for predicting liposuction efficacy in patients with breast cancer-related lymphedema and further implemented it as a web-based calculator. The support vector machine model demonstrated favorable predictive performance, stability, and clinical applicability. To our knowledge, this is the first machine learning model specifically designed to predict postoperative response to liposuction in patients with BCRL, rather than the risk of lymphedema occurrence. The model may assist clinicians in preoperative risk stratification, patient counseling, and surgical decision-making, thereby providing a useful basis for optimizing management strategies and individualized treatment planning in BCRL.

## Data Availability

The original contributions presented in the study are included in the article/supplementary material. Further inquiries can be directed to the corresponding authors.

## References

[1] Penn IW Chang YC Chuang E Chen CM Chung CF Kuo CY . Risk factors and prediction model for persistent breast-cancer-related lymphedema: a 5-year cohort study. Support Care Cancer. (2019) 27:991–1000. doi: 10.1007/s00520-018-4388-6 30105666 PMC6373263

[2] DiSipio T Rye S Newman B Hayes S . Incidence of unilateral arm lymphoedema after breast cancer: a systematic review and meta-analysis. Lancet Oncol. (2013) 14:500–15. doi: 10.1016/s1470-2045(13)70076-7 23540561

[3] Hinrichs CS Watroba NL Rezaishiraz H Giese W Hurd T Fassl KA . Lymphedema secondary to postmastectomy radiation: incidence and risk factors. Ann Surg Oncol. (2004) 11:573–80. doi: 10.1245/aso.2004.04.017 15172932

[4] Leung N Furniss D Giele H . Modern surgical management of breast cancer therapy related upper limb and breast lymphoedema. Maturitas. (2015) 80:384–90. doi: 10.1016/j.maturitas.2015.01.012 25747119

[5] Donahue PMC MacKenzie A Filipovic A Koelmeyer L . Advances in the prevention and treatment of breast cancer-related lymphedema. Breast Cancer Res Treat. (2023) 200:1–14. doi: 10.1007/s10549-023-06947-7 37103598 PMC10224871

[6] Brorson H . Liposuction in lymphedema treatment. J Reconstr Microsurg. (2016) 32:56–65. doi: 10.1055/s-0035-1549158 25893630

[7] Chen J Feng X Zhou Y Wang Y Xiao S Deng C . Outcomes after liposuction-based treatment of lymphedema: a systematic review and meta-analysis. Front Oncol. (2025) 15:1651472. doi: 10.3389/fonc.2025.1651472 41383504 PMC12689340

[8] Wei X Lu Q Jin S Li F Zhao Q Cui Y . Developing and validating a prediction model for lymphedema detection in breast cancer survivors. Eur J Oncol Nurs. (2021) 54:102023. doi: 10.1016/j.ejon.2021.102023 34500318

[9] Kwan JYY Famiyeh P Su J Xu W Kwan BYM Jones JM . Development and validation of a risk model for breast cancer-related lymphedema. JAMA Netw Open. (2020) 3:e2024373. doi: 10.1001/jamanetworkopen.2020.24373 33175175 PMC7658732

[10] Li Y Guan L Ning C Zhang P Zhao Y Liu Q . Machine learning-based models to predict one-year mortality among Chinese older patients with coronary artery disease combined with impaired glucose tolerance or diabetes mellitus. Cardiovasc Diabetol. (2023) 22:139. doi: 10.1186/s12933-023-01854-z 37316853 PMC10268354

[11] Luo W Phung D Tran T Gupta S Rana S Karmakar C . Guidelines for developing and reporting machine learning predictive models in biomedical research: a multidisciplinary view. J Med Internet Res. (2016) 18:e323. doi: 10.2196/jmir.5870 27986644 PMC5238707

[12] Sitzia J . Volume measurement in lymphoedema treatment: examination of formulae. Eur J Cancer Care (Engl). (1995) 4:11–6. doi: 10.1111/j.1365-2354.1995.tb00047.x 7620649

[13] Armer JM Ballman KV McCall L Armer NC Sun Y Udmuangpia T . Lymphedema symptoms and limb measurement changes in breast cancer survivors treated with neoadjuvant chemotherapy and axillary dissection: results of American College of Surgeons Oncology Group (ACOSOG) Z1071 (Alliance) substudy. Support Care Cancer. (2019) 27:495–503. doi: 10.1007/s00520-018-4334-7 29980907 PMC6342501

[14] Burke HB . Transparent reporting of a multivariable prediction model for individual prognosis or diagnosis (TRIPOD). Ann Intern Med. (2015) 162:735. doi: 10.7326/l15-5093 25984856

[15] Armer JM Stewart BR . A comparison of four diagnostic criteria for lymphedema in a post-breast cancer population. Lymphat Res Biol. (2005) 3:208–17. doi: 10.1089/lrb.2005.3.208 16379589

[16] Li P Zhao Z Sun Y Xia S Shen W . The prognostic effect and mechanism of erysipelas in cancer-associated lymphedema. Sci Rep. (2025) 15:5518. doi: 10.1038/s41598-025-90200-2 39953144 PMC11828868

[17] Bundred N Foden P Todd C Morris J Watterson D Purushotham A . Increases in arm volume predict lymphoedema and quality of life deficits after axillary surgery: a prospective cohort study. Br J Cancer. (2020) 123:17–25. doi: 10.1038/s41416-020-0844-4 32362658 PMC7341763

[18] Pudjihartono N Fadason T Kempa-Liehr AW O'Sullivan JM . A review of feature selection methods for machine learning-based disease risk prediction. Front Bioinform. (2022) 2:927312. doi: 10.3389/fbinf.2022.927312 36304293 PMC9580915

[19] Han J Gondro C Reid K Steibel JP . Heuristic hyperparameter optimization of deep learning models for genomic prediction. G3 (Bethesda). (2021) 11:jkab032. doi: 10.1093/g3journal/jkab032 33993261 PMC8495939

[20] Lundberg SM Erion G Chen H DeGrave A Prutkin JM Nair B . From local explanations to global understanding with explainable AI for trees. Nat Mach Intell. (2020) 2:56–67. doi: 10.1038/s42256-019-0138-9 32607472 PMC7326367

[21] Wilkinson L Gathani T . Understanding breast cancer as a global health concern. Br J Radiol. (2022) 95:20211033. doi: 10.1259/bjr.20211033 34905391 PMC8822551

[22] Li T Mello-Thoms C Brennan PC . Descriptive epidemiology of breast cancer in China: incidence, mortality, survival and prevalence. Breast Cancer Res Treat. (2016) 159:395–406. doi: 10.1007/s10549-016-3947-0 27562585

[23] Penn IW Chang YC Chuang E Chen CM Chung CF Kuo CY . Risk factors and prediction model for persistent breast-cancer-related lymphedema: a 5-year cohort study. Support Care Cancer. (2019) 27:991–1000. doi: 10.1007/s00520-018-4388-6 30105666 PMC6373263

[24] Rockson SG Keeley V Kilbreath S Szuba A Towers A . Cancer-associated secondary lymphoedema. Nat Rev Dis Primers. (2019) 5:22. doi: 10.1038/s41572-019-0072-5 30923312

[25] Dylke ES Ward LC . Three decades of bioelectrical impedance spectroscopy in lymphedema assessment: an historical perspective. Lymphat Res Biol. (2021) 19:206–14. doi: 10.1089/lrb.2020.0085 33232645

[26] Jeon H Kim DY Park SW Lee BS Kim D Han HW . Biomarkers in lymphedema assessment: integrating elastography and muti-frequency bioimpedance analysis. biomark Med. (2024) 18:983–93. doi: 10.1080/17520363.2024.2415283 39445460 PMC11633427

[27] Otsuka W Yoshida S Taketomi N Orihashi Y Koshima I . The role of bioelectrical impedance analysis in predicting secondary surgical interventions for lymphedema. J Clin Med. (2025) 14:2151. doi: 10.3390/jcm14072151 40217602 PMC11989653

[28] Yasunaga Y Kinjo Y Yanagisawa D Yuzuriha S Kondoh S . Changes in intracellular water volume after leg lymphedema onset and lymphaticovenular anastomosis as its surgical intervention. J Vasc Surg Venous Lymphat Disord. (2023) 11:1243–52. doi: 10.1016/j.jvsv.2023.07.010 37536561

[29] Li P Zhao Z Sun Y Xia S Shen W . The prognostic effect and mechanism of erysipelas in cancer-associated lymphedema. Sci Rep. (2025) 15:5518. doi: 10.1038/s41598-025-90200-2 39953144 PMC11828868

[30] Zampell JC Aschen S Weitman ES Yan A Elhadad S De Brot Andrade M . Regulation of adipogenesis by lymphatic fluid stasis: part I. Adipogenesis, fibrosis, and inflammation. Plast Reconstr Surg. (2012) 129:825–34. doi: 10.1097/prs.0b013e3182450b47 22456354 PMC3433726

[31] Mehrara BJ Greene AK . Lymphedema and obesity: is there a link? Plast Reconstr Surg. (2014) 134:154e–60e. doi: 10.1016/b978-0-323-65381-7.00044-7 25028830 PMC4393748

[32] Wei X Lu Q Jin S Li F Zhao Q Cui Y . Developing and validating a prediction model for lymphedema detection in breast cancer survivors. Eur J Oncol Nurs. (2021) 54:102023. doi: 10.1016/j.ejon.2021.102023 34500318

